# Host–bacteria interactions: ecological and evolutionary insights from ancient, professional endosymbionts

**DOI:** 10.1093/femsre/fuae021

**Published:** 2024-07-30

**Authors:** Zélia Bontemps, Kiran Paranjape, Lionel Guy

**Affiliations:** Department of Medical Biochemistry and Microbiology, Science for Life Laboratory, Uppsala University, 75237 Uppsala, Sweden; Department of Medical Biochemistry and Microbiology, Science for Life Laboratory, Uppsala University, 75237 Uppsala, Sweden; Department of Medical Biochemistry and Microbiology, Science for Life Laboratory, Uppsala University, 75237 Uppsala, Sweden

**Keywords:** endosymbionts, microbial ecology, evolution, host–bacteria interactions, microbial interactions

## Abstract

Interactions between eukaryotic hosts and their bacterial symbionts drive key ecological and evolutionary processes, from regulating ecosystems to the evolution of complex molecular machines and processes. Over time, endosymbionts generally evolve reduced genomes, and their relationship with their host tends to stabilize. However, host–bacteria relationships may be heavily influenced by environmental changes. Here, we review these effects on one of the most ancient and diverse endosymbiotic groups, formed by—among others—*Legionellales, Francisellaceae*, and *Piscirickettsiaceae*. This group is referred to as Deep-branching Intracellular *Gammaproteobacteria* (DIG), whose last common ancestor presumably emerged about 2 Ga ago. We show that DIGs are globally distributed, but generally at very low abundance, and are mainly identified in aquatic biomes. Most DIGs harbour a type IVB secretion system, critical for host-adaptation, but its structure and composition vary. Finally, we review the different types of microbial interactions that can occur in diverse environments, with direct or indirect effects on DIG populations. The increased use of omics technologies on environmental samples will allow a better understanding of host–bacterial interactions and help unravel the definition of DIGs as a group from an ecological, molecular, and evolutionary perspective.

## Teaser

Deep-branching Intracellular *Gammaproteobacteria* (DIG), among the oldest groups of professional endosymbionts, are widely distributed around the world, possess specific molecular mechanisms, and also have the ability to interact with other microorganisms in environments that determine the nature of their interactions with their hosts along the mutualism–parasitism continuum.

## Introduction

Diverse organisms live together, and by doing so become more than they are as individuals: they form symbioses (Bary 1879). Symbioses are diverse, ubiquitous in nature, and represent major driving forces of evolutionary change (Bennett and Moran 2015, Oliver and Russell 2016). They create conditions favourable to the emergence of complex life forms along the mutualism–parasitism continuum, allowing for expansion into previously inaccessible ecological niches (Toft and Andersson 2010, Skelton et al. 2016, Sudakaran et al. 2017). Interactions along the mutualism–parasitism continuum include (i) mutualism, where organisms jointly cooperate, (ii) commensalism, where one partner benefits with no effect to the other, and (iii) parasitism, where only one of the partners benefits at the expense of the other (Parmentier and Michel 2013). Symbionts can be either intracellular (endosymbionts), extracellular (ectosymbionts), or can be tightly associated with the host (such as episymbionts). Endosymbionts, which are the focus of this review, can be facultative or obligate, depending on whether the symbionts can survive extended periods of time outside of their host (Toft and Andersson 2010). By definition, endosymbionts live intracellularly within a host and may reside in the cytoplasm, inside vacuoles, or even inside the host nucleus or mitochondria.

Endosymbioses are at the origin of some of the most important eukaryotic innovations. It is widely accepted that mitochondria and plastids, in early eukaryotes, were derived from an alphaproteobacterial endosymbiont and a cyanobacterial endosymbiont, respectively (Sagan 1967, Huang and Gogarten 2007, Gray 2012, Martijn et al. 2018, Sibbald and Archibald 2020). Besides these, many other bacterial groups have been associated with eukaryotes, some of them for over a billion years (Toft and Andersson 2010). The most diverse, successful, and ancient endosymbiont lineages closely associated with eukaryotes are found within *Alphaproteobacteria, Gammaproteobacteria*, and *Chlamydiae* (Köstlbacher et al. 2021, Wang and Luo 2021, Hugoson et al. 2022). Members of these lineages can be described as ancient professional endosymbionts (Husnik et al. 2021) because they have established long-term symbiotic relationships, allowing them to accumulate intricate adaptations with their hosts through coevolution over hundreds of millions of years. Endosymbionts interact with their hosts in mutualistic, commensal, or pathogenic manners, and include pathogens that are significant causes of disease in human, livestock and crop (Duron et al. 2018a, Dharamshi 2021, Schön et al. 2022). Specific molecular mechanisms are well-described for certain ancient professional endosymbionts, mostly within *Alphaproteobacteria* and *Chlamydiae*. Indeed, *Rickettsiales* use several known factors to interact with their hosts, including a type IV secretion system, effector proteins that manipulate the host cell, and an Adenosine triphosphate (ATP) / Adenosine diphosphate (ADP) translocase, which facilitates energy parasitism by exchanging ATP for endogenous ADP from the host cell (Schön et al. 2022). In general, secretion systems are crucial to host–symbiont interactions across the mutualism–parasitism continuum, spread by horizontal transfer and evolve through co-option, neofunctionalization, speciation, and *de novo* acquisition of single or multiple proteins at a time (Denise et al. 2020). Secretion systems, thus, provide endosymbionts with various specific functions, e.g. facilitating cell adhesion, injecting effectors and virulence factors during host infection, or excreting toxic compounds (Segal et al. 2005, Costa et al. 2015). The study of ancient endosymbionts and their host-interaction systems is invaluable for elucidating the evolutionary processes and the specific molecular and ecological mechanisms underlying host–endosymbiont interactions, along the mutualism–parasitism continuum. Some endosymbiont groups, such as the ones mentioned above, are well-reviewed (Köstlbacher et al. 2021, Wang and Luo 2021, Castelli et al. 2022, Dharamshi et al. 2023) but much less is known about the—potentially—oldest one, which we refer to as the Deep-branching Intracellular *Gammaproteobacteria* (DIG).

**Figure 1.**
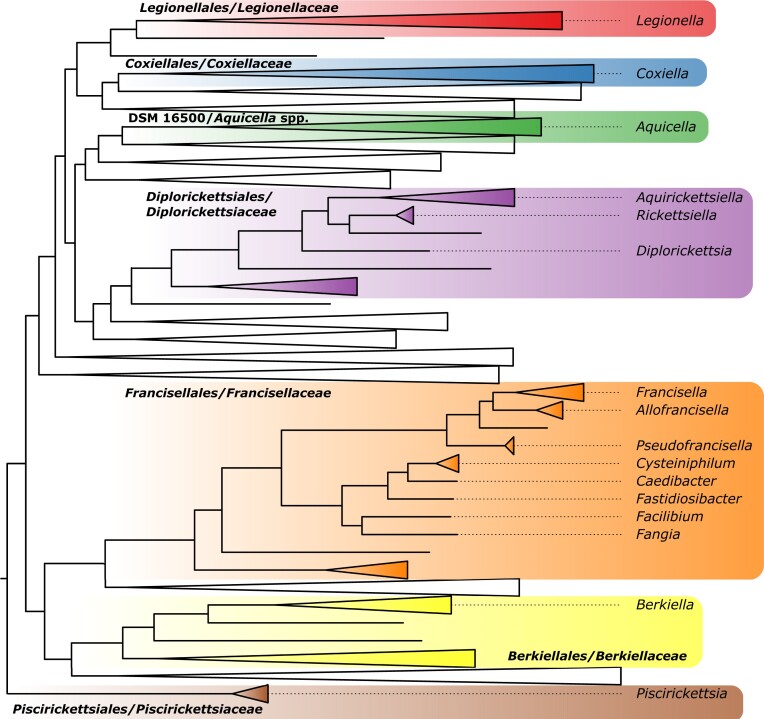
Simplified phylogeny of DIGs. The tree is based on the bac120 tree obtained from GTDB (Parks et al. 2022), release 214. The tree was pruned using Newick utilities (Junier and Zdobnov 2010) to keep only the subtree encompassing the last common ancestor (LCA) of the DIG classes (as defined by GTDB) *Legionellales, Coxiellales*, DSM1500 (*Aquicella* spp.), *Diplorickettsiales, Francisellales, Berkiellales*, and *Piscirickettsiales*. All 337 descendants of the DIG LCA were kept, but larger groups were collapsed for readability. On the tree, classes are highlighted according to their placement in GTDB.

DIGs are defined as the *Legionellales*, traditionally divided in two families, *Legionellaceae* and *Coxiellaceae*, and several related intracellular groups. The *Legionellales* order harbours a wide ecological diversity of genera with: (i) facultative intracellular pathogens of amoebae such as *Aquicella* and *Legionella* (*Legionella* are also accidental human pathogens, causing Legionnaires’ disease); (ii) obligate intracellular pathogens, such as *Coxiella burnetii*, the agent of Q-fever; (iii) facultative intracellular symbionts of arthropods such as ‘*Ca*. Rickettsiella viridis (Tsuchida et al. 2010), and (iv) even an obligate mutualistic endosymbiont of a ciliate, in which the endosymbiont has replaced the mitochondrion (Graf et al. 2021). Beyond *Legionellales*, DIGs also include *Francisellaceae* (including mammalian pathogens like *F. tularensis*), *Fastidiosibacteraceae* (such as *Paramecium* endosymbionts from marine seawater environment), and *Piscirickettsiaceae* (containing so far only one intracellular genus, *Piscirickettsia*, a fish pathogen) (Duron et al. 2018b, Barril 2022, Xiao et al. 2018, Fryer and Hedrick 2003). These groups, named above as in the traditional taxonomies (e.g. at NCBI or through LPSN) (Parte et al. 2020), have been reclassified as orders in the genome-based taxonomy developed by Genome Taxonomy Database (GTDB) (Parks et al. 2022). Hereafter and wherever possible, we will adopt the latter classification (Table 1), among others because the GTDB tree shows the main DIG classes as monophyletic (Fig. 1). Furthermore, one of the landmarks of DIGs (although there are notable exceptions, like the *Francisella* genus) is the possession of a type IVB secretion system (T4BSS). This secretion system facilitates symbiotic interactions all across the parasite–mutualism continuum, by injecting effector proteins into the host cytoplasm (Costa et al. 2015, Hugoson et al. 2022).

**Table 1.** Nonexhaustive list of taxa in the group Deep Intracellular *Gammaproteobacteria* and their related host/environment.

Taxonomy (GTDB)					Host/environment	Endosymbiont status	References	Taxonomy (NCBI)	
Class	Order	Family	Genus	Species				Order	Family
*Gammaproteobacteria*	DSM-16500	DSM-16500	*Aquicella*	*A. lusitana*	Freshwater environment	Facultative	Santos et al. (2003)	*Legionellales*	*Coxiellaceae*
				*A. siphonis*	Freshwater environment	Facultative	Santos et al. (2003)		
	?	?	*Ca*. Pokemonas	*Ca*. Pokemonas kadabra and *Ca*. P. abra	Rhizarian amoebae	Facultative	Solbach et al. (2021)		
	*Berkiellales*	*Berkiellaceae*	*Berkiella*	*Ca*. Berkiella aquae	Fresh water species and biofilms	Facultative	Mehari et al. (2016)		
				*Ca*. Berkiella cookevillensis	Fresh water species and biofilms	Facultative	Mehari et al. (2016)		
				*Ca*. Fiscibacter pecunis	Rhizarian amoebae	Facultative	Solbach et al. (2021)		
				*Ca*. Rhogoubacter	Rhizarian amoebae	Facultative	Solbach et al. (2021)		
				*Ca*. Nucleophilum amoebae	Marine amoebae	Facultative	Solbach et al. (2021)		
				*Ca*. Ovatusbacter abovo	Amoeboid opisthokont	Facultative	Dirren and Posh (2016)		
	*Coxiellales*	*Coxiellaceae*	*Coxiella*	*Ca*. Coxiella mudrowiae	Ticks	Obligate	Gottlieb et al. (2015)		
				*Ca*. Occultobacter vannellae	Testate amoebae	Obligate	Schulz et al. (2015)		
				*Ca*. Cochliophilus	Freshwater testate amoebae	Obligate	Schulz et al. (2015)		
				*C. burnetti*	Domestic ruminants	Obligate	Duron et al. (2015)		
				*C. cheraxi*	Crayfish	Obligate	Davidovich et al. (2022)		
	*Diplorickettsiales*	*Diplorickettsiaceae*	*Aquiricketsiella*	*Rickettsiella grylli*	Aquatic and terrestrial arthropods	Obligate	Leclerque (2008)		
				Aquirickettsiella gammarie	Freshwater crustaceans	Facultative	Bojko et al. (2018)		
			*Diplorickettsia*	*Rickettsiella massiliensis*	Ticks	Obligate	Leclerque and Kleespies (2008)		
			*Rickettsiella*	*Ca*. Rickettsiella viridis	Aphids	Facultative	Tsuchida et al. (2014)		
				*R. agriotidis*	Neoptera	Obligate	Leclerque et al. (2011)		
				*R. ixodidis*	Ixodidae	Obligate	Leclerque and Kleespies (2012)		
				Other *Rickettsiella* species	Diverse arthropod species	Obligate	Bouchon et al. (2011)		
	*Legionellales*	*Legionellaceae*	*Legionella*	*L. dumoffi*	Fresh water species and biofilms	Facultative	Qin et al. (2012)		*Legionellaceae*
				*L. antartica*	Fresh water	Facultative	Shimada et al. (2021)		
				*L. longbeachae*	Amoebae and compost dust	Facultative	Whiley and Bentham (2011)		
				*L. massiliensis*	Fresh water species and biofilms	Facultative	Campocasso et al. (2012)		
				*L. micdadei*	Water and soil	Facultative	Lachant and Prasad (2015)		
				*L. pneumophila*	Amoebae and biofilms in freshwater environments	Facultative	Goncalves et al. (2021)		
				*L. polyplacis*	Rodent lice	Obligate	Ríhová et al. (2017)		
				*L. steelei*	Water	Facultative	Edelstein et al. (2012)		
				Other *Legionella* species	Amoebae and biofilms in freshwater environments	Facultative	Khodr et al. (2016)		
		?	?	*Ca*. Azoamicus ciliaticola	Anaerobic ciliate	Obligate	Graf et al. (2021)		
	*Francisellales*	*Francisellaceae*	*Fastidiosibacter*	*F. lacustris*	Fresh water	Free living?	Xiao et al. (2018)	*Thiotrichales*	*Fastidiosibacteraceae*
			*Facilibium*	*F. subflavum*	Fresh water	Free living?	Xiao et al. (2018 )		
			*Cysteiniphilum*	*Cysteiniphilum* spp.	Fresh water	Free living?	Xiao et al. (2018)		
			*Fangia*	*F. hongkongensis*	Coastal seawater	Free living?	Sjodin et al. (2012)		
			*Caedibacter*	*C. taeniospiralis*	Marine ciliates	Obligate	Grosser et al. (2018)		
			*Allofrancisella*	*A. frigidaquae*	Water cooling system	Facultative?	Qu et al. (2016)		*Francisellaceae*
				*A. guangzhouensis*	Air-conditioning systems	Facultative?	Qu et al. (2013)		
				*A. inopinata*	Water cooling system	Facultative?	Qu et al. (2016)		
			*Francisella*	*Ca*. Francisella endociliophora	Marine ciliates	Facultative	Sjodin et al. (2012)		
				*F. orientalis*	Freshwater, seawater, and fish	Facultative	Hennebique et al. (2019)		
				*F. adeliensis*	Antartic marine ciliate	Facultative	Vallesi et al. (2019)		
				*F. tularensis*	Biofilms, amoebae, and mosquito larvae	Facultative	Hennebique et al. (2019)		
				*F. ulginis*	Water and aerosols	Facultative	Petersen et al. (2009)		
				Other *Francisella* species	Freshwater and seawater environments	Facultative	Petersen et al. (2009), Li et al. (2020)		
			*Pseudofrancisella*	*P. aestuarii*	Estuarine seawater	Facultative?	Zheng et al. (2019)		
	*Piscirickettsiales*	*Piscirickettsiaceae*	*Piscirickettsia*	*P. litoralis*	Seawater species and biofilms	Facultative?	Wan et al. (2016)		*Piscirickettsiaceae*
				*P. salmonis*	Isopodes species and seaweater	Facultative	Ramirez et al. (2015)		

This article reviews host–bacteria interactions, with a focus on ecological and evolutionary insights from DIG bacteria. The environmental distribution of these ancient professional bacterial endosymbionts is reviewed in section - Environmental distribution of DIGs, along with their diversity in different biomes. Their molecular features are then discussed in section - Impact of molecular mechanisms on the mutualism–parasitism continuum of DIGs, including their impact on the placement along the mutualistic–pathogenic continuum. Finally, the interactions between endosymbionts and other microorganisms present in their host are reported in section - Influence of the microbiota—interaction with other bacteria and endosymbionts.

## Environmental distribution of DIGs

Growing DIGs in laboratory settings is difficult, due to their intracellular lifestyle and dependence on their host (Table 1). Currently, out of the potential hundreds of genera of DIGs (Graells et al. 2018), only species of the genera *Legionella, Aquicella, Coxiella, Piscirickettsia, Fangia*, and *Francisella* can be grown axenically (Santos et al. 2003, Omsland 2012, Martínez et al. 2018, Xiao et al. 2018, Vallesi et al. 2019). Fastidious organisms often cannot be sequenced with traditional genomic techniques, making metagenomics the method of choice for exploring their diversity. A previous study, which examined large amounts of metabarcoding data, found that *Legionellales* are diverse and ubiquitous (both geographically and environmentally), but at very low abundance (about 0.1%) (Graells et al. 2018). Shotgun metagenomics is providing a better view of the distribution, diversity, and functional potential of DIGs. Analysis of DIGs through metagenomic datasets are crucial to better understanding potential vectors for emerging pathogens in relation to global environmental changes (Ranjan et al. 2016).

In this section, we surveyed the geographic and environmental distribution of DIGs, reviewing publicly available metagenomic data. Briefly, data and metadata were collected for all samples present on MGnify using the available Application Programming Interface (API) and samples with at least one 16S rRNA read attributed to DIGs were retained for analysis (*n* = 5605) (Gurbich et al. 2023). To test a more conservative approach, cut-offs of 3 reads (Supplementary Figs 1–4) and 10 reads were applied and the number of positive samples decreased (−27% and −53%, respectively). As most DIGs are rare (Graells et al. 2018), such a decrease is expected with a 3-, respectively 10-fold increase in the cut-off. Diversity, geographic distribution, and the effect of environmental factors (temperature, depth, and pH) on DIG abundance were gathered and visualized using Python, including the package mgtoolkit, and R, including the packages ggplot2, tidyverse, ggpubr, maptools, ggsn, scales, and phyloseq (Van Rossum and Drake 2009, Baquero 2019, R Core Team 2022, Wickham et al. 2022, Bivand et al. 2023, Kassambara 2023, McMurdie and Holmes 2023, Ranathunga 2023, Wickham and RStudio 2023, Wickham et al. 2023).

**Figure 2.**
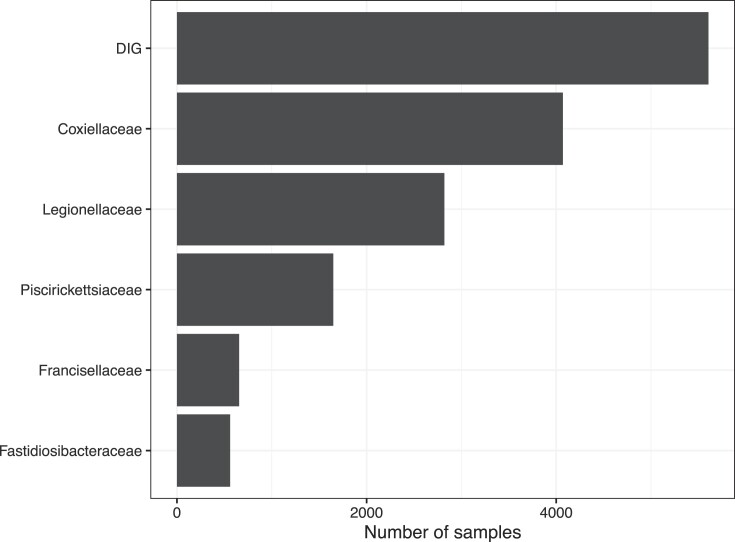
Global distribution of metagenomic samples from public databases and sample distribution across DIG families. MGnify has used different taxonomies in the different versions of its pipeline, but most analyses were performed with the SILVA database (prior version 138), which mostly reflects the traditional taxonomy. At family level, the discrepancies are minor, but the genera from *Fastidiosibacteraceae* are included in *Francisellaceae* in the GTDB taxonomy.

This survey identified 5605 metagenomes containing at least one DIG-related sequence, representing 28.9% of all datasets publicly available from the European Bioinformatics Institute (Fig. 2). At the family level, *Coxiellaceae* were found in most samples (*n* = 4070, 41.7%), followed by *Legionellaceae* (*n* = 2820, 28.9%), *Piscirickettsiaceae* (*n* = 1649, 16.9%), *Francisellaceae* (*n* = 656, 6.7%), and *Fastidiosibacteraceae* (*n* = 561, 5.8%).

### Geographical distribution

**Figure 3.**
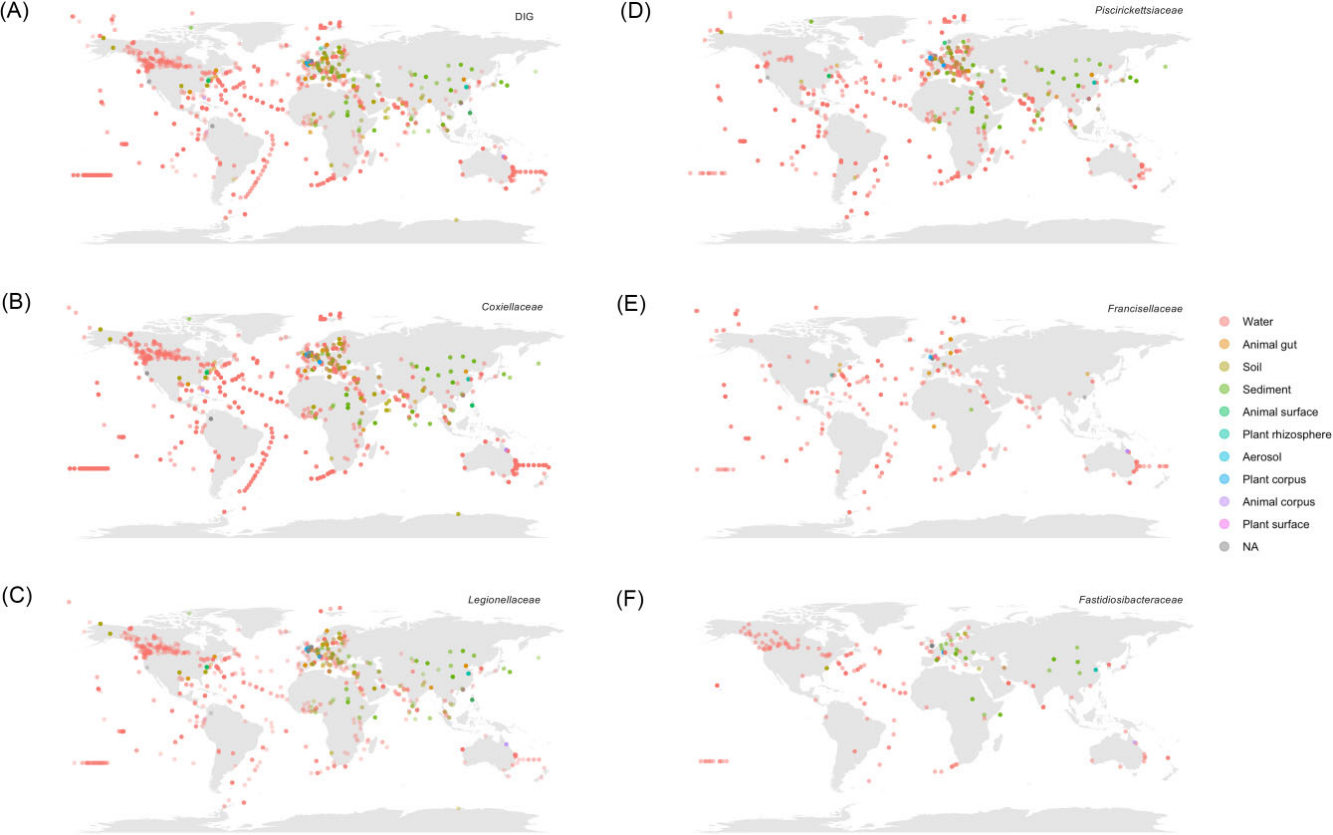
Geographical distribution of DIGs in various environments. Each panel represents one of the five selected DIG families. The sampling location is represented with a dot. Samples are coloured according to the biome of origin. (A) DIG, (B) *Coxiellaceae*, (C) *Legionellaceae*, (D) *Piscirickettsiaceae*, (E) *Francisellaceae*, and (F) *Fastidiosibacteraceae*.

DIGs are globally distributed across the world, with a few exceptions (Fig. 3). This is particularly true for the *Coxiellaceae, Legionellaceae*, and *Piscirickettsiaceae*: all continents harbour at least one sequence affiliated to them. This distribution suggests that these families are generalist taxa, i.e. taxa with a cosmopolitan distribution and metabolically active according to their level of tolerance to environmental conditions (Kilroy et al. 2007, Cox et al. 2019). The *Legionellaceae* and *Coxiellaceae* datasets are dominated by two types of genera, *Legionella* and *Coxiella* (>65%). These two genera are well-represented in the databases, and are responsible for major public health problems on five continents (Celina and Cerný 2022, Lockwood et al. 2022). Cases of Legionnaires’ disease and Q fever have sharply increased over the last two decades, with an 8–10-fold increase of Legionnaires’ disease in Europe and a 2–3-fold increase of Q fever in the United States (Committee on Management of Legionella in Water System 2019, CDC 2021). In general, amoebae and ticks—both globally distributed—are the most well known hosts for the *Legionellaceae* and *Coxiellaceae*, respectively (Duron et al. 2017, Celina and Cerný 2022). Unsurprisingly, *Legionellaceae* and *Coxiellaceae* are the most geographically widespread families, presumably due to the global distribution of their hosts and the increased outbreak monitoring and tracking (Felice et al. 2019).

*Piscirickettsiaceae* is also widely distributed (Fig. 3D), with a high concentration of samples that were found in the Atlantic and Pacific Oceans. Many salmon farms are located in these oceans, which is a likely explanation for the presence of reads attributed to *Piscirickettsia salmonis*, the agent of piscirickettsiosis (an infectious salmon disease) (Rozas-Serri 2022).

*Fastidiosibacteraceae* is globally distributed, although to a lesser degree than the former families (Fig. 3F). They are not found close to the poles (Arctic and Antarctic). Moreover, compared to the other DIG families, there are fewer positive samples in the Southern Hemisphere (<35%) (Fig. 3F). Only five described genera are part of this family, and all originate from aquatic environments from five continents (e.g. *Fangia hongkongensis* isolated from Asia) (Xiao et al. 2018). In addition, *Caedibacter taeniospiralis* reproduces in the globally distributed protist *Paramecium caudatum* (Beier et al. 2002, Krenek et al. 2012). However, the lower abundance of this family in our dataset is likely due to the lack of documentation and the small number of species it contains, and is unlikely to represent the reality of its geographical distribution.

*Francisellaceae* is also distributed worldwide, with an especially important presence in salt water environments, particularly in the Atlantic and Pacific Oceans (Fig. 3E). Several *Francisellaceae* species, e.g. *Francisella noatunensis* (Colquhoun and Duodu 2011), *F. piscicida* (Ottem et al. 2008) are fish pathogens, while others, e.g. *F. salimarina* (Li et al. 2020) and *Pseudofrancisella aestuarii* (Zheng et al. 2019) were isolated from estuaries. Among those found on land, the species *F. tularensis*, responsible for the zoonosis tularemia, is spread over long distances by its hosts, such as ticks and mosquitoes (Lundström et al. 2011, Hennebique et al. 2019).

### Biomes

**Figure 4.**
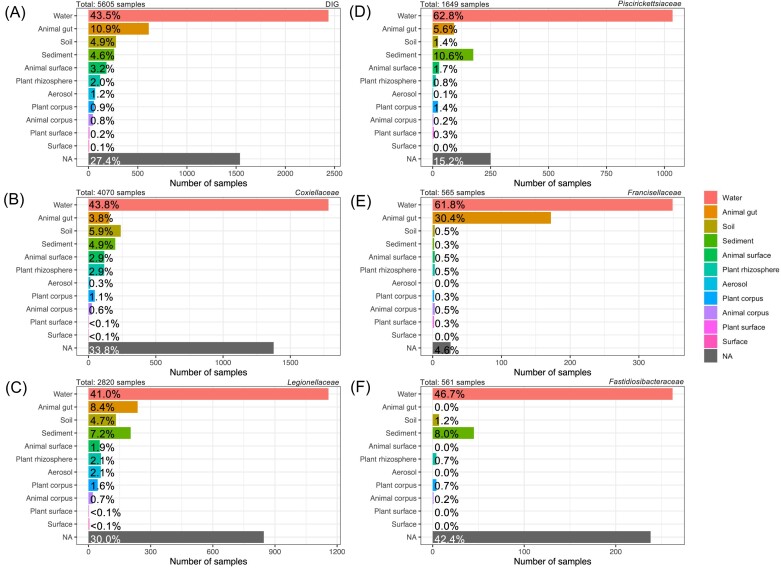
Number of DIG-positive samples across various biomes for DIGs (A), *Coxiellaceae* (B), *Legionellaceae* (C), *Piscirickettsiaceae* (D), *Francisellaceae* (E), and *Fastidiosibacteraceae* (F).

DIGs are present in all major biome categories, as defined by the ontology from the Earth Microbiome project (EMP ontology) (Thompson et al. 2017), in varying proportions. As expected, water samples displayed the highest share of positive samples (43%), but other biomes also had a high proportion of samples positive for DIGs: animal corpus (mostly gut samples), 10%; soil, 4.9%; and sediments, 4.6%. A total of 10% of the biomes that could not be classified as above were also positive for DIGs. (Fig. 4A).

Among all samples where DIG families are present, about half (49.5% on average over the five families) are, on average, from the aquatic biome (Fig. 4). Despite being present in many aquatic samples, DIGs are mostly there in low abundance, less than 0.01%. Of the more abundant DIG family taxa, *Piscirickettsiaceae* and *Legionnnellaceae* are found in higher abundance in the latter ecosystems. While frequently identified in freshwater or soil samples, or within their hosts, *Coxiellaceae, Legionellaceae, Piscirickettsiaceae*, and *Francisellaceae* have been, more recently, also found in salt waters (Tsao et al. 2017, Hastuti et al. 2022, Bergman et al. 2023, Eriksson et al. 2023). The genus *Francisella* (*Francisellaceae*) is found in extreme aquatic ecosystems, such as deep-sea hydrothermal vents in the Pacific and Atlantic oceans, where it can play a role in sulphur oxidation (Sylvan et al. 2012, Ding et al. 2017). Certain microorganisms of the family *Piscirickettsiaceae* are commonly found in hypersaline, sulphur-rich, or soda lakes (generally at low abundances <0.5%), supporting the hypothesis of an ecological role for these organisms in sulphur and methanogenesis cycles (Borin et al. 2009, Paul et al. 2016). The *Fastidiosibacteraceae* family is poorly studied, and all isolates currently available in databases are exclusively from lake ecosystems (Xiao et al. 2018, Salam et al. 2019); for example the species *Fastidiosibacter lacustris* was isolated from a water sample from Lake Haizhu in China (Xiao et al. 2018, Brunet et al. 2021, Davidovich et al. 2022). Sewage treatment plants are increasingly considered as sources of pathogens and species of *Legionellaceae* and *Coxiellaceae* have been identified in these environments (Schets et al. 2013, van den Berg et al. 2023). In general, aerobic biological water treatment systems provide an optimal environment for the growth of these microorganisms due to high concentrations of organic nitrogen and oxygen, ideal temperatures, and the presence of protozoa (Caicedo et al. 2019). The survival of some of these endosymbionts in aquatic ecosystems may be favoured by their hosts; indeed, amoebae are common hosts for *Legionellaceae, Coxiellaceae*, and *Francisellaceae* (Duron et al. 2018a, Hennebique et al. 2019, Price and Abu Kwaik 2021, Solbach et al. 2021). In 2021, a study looked in detail at the long-term interactions of the tularemia pathogens *F. tularensis* subsp*. holarctica, F. novicida*, or *F. philomiragi*a with amoebae of the *Acanthamoeba* species and showed that amoebae are likely to promote the survival of *Francisella* in aquatic environments, including the tularemia pathogen *F. tularensis* (Hennebique et al. 2021).

Several DIGs have aquatic metazoans as hosts, which may explain their high prevalence both in aquatic and animal gut biome categories (on average 8.8%, Fig. 4). It is particularly apparent for *Francisellaceae*, present in 26% of the animal gut samples (Fig. 4E). This might be explained by the recurrent isolation of *F. tularensis* from the digestive systems of mosquitoes, bedbugs, lice, flies, and ticks (Telford and Goethert 2020). *Francisella tularensis* survives the harsh, acidic condition found in the insect gut tracts, mostly by invading erythrocytes that ticks ingest during a blood meal (Schmitt et al. 2017). In addition to protection from low pH conditions, erythrocytes provide haemoglobin, which can help counteract oxidative stress, and heme, which serves as an iron source necessary for *F. tularensis* survival. The high prevalence of *Piscirickettsiaceae* and *Coxiellaceae* both in aquatic and animal gut biome categories might also be caused by their metazoan hosts.  *Piscirickettsia salmonis* causes piscirickettsiosis, a disease affecting farmed salmon, trout and sea bass. Piscirickettsiosis is a systemic infection characterized by colonization of multiple organs including the kidney, liver, spleen, intestine, brain, ovary, and gills of salmon (Aravena et al. 2020), typically resulting in mortality rates between 10% and 30%. In addition to ticks, some *Coxiellaceae* species also colonize the gut of other metazoans (3.8% of samples, Fig. 4B). For example, *Coxiella cheraxi*, causes rickettsiosis in the red-legged crayfish (*Cherax quadricarinatus*). *Coxiella cheraxi* can colonize the midgut of its host and cause 22%–80% mortality in naturally infected crayfish (Davidovich et al. 2022).

Sediment samples accounted for an average of 6% of the samples positive for DIGs (Fig. 4). For *Piscirickettsiaceae* and *Fastidiosibacteraceae*, it amounted to 10.6% and 8.0% of samples, respectively (Fig. 4D and F). Several studies have identified *Piscirickettsiaceae* in a variety of sediments, including coastal, riverine, deep, and estuarine sediments (Cleary et al. 2017, Xie et al. 2018, Sonthiphand et al. 2023). *Fastidiosibacteraceae* are generally isolated from lake biomes, where sediment samples are collected, which partly explains their high prevalence in this biome type. During sedimentation and the passage of overlaying water at the sediment–water interface, the number of bacteria increased by 3–5 orders of magnitude, perhaps making them easier to detect in this biome (Wetzel 2001). Amoebae, a common host of *Coxiellaceae* and *Legionellaceae*, are identified in most biomes, including sediments. It may explain why 4.9% and 7.1% of samples from this biome category are positive for theses two families, respectively: e.g. *Legionella* are found in tank sediments, (Lu et al. 2015) and *Coxiella* in water plant system sediments, (Corsaro et al. 2010). The DIGs are also found in sediments from mines and anthropized caves. Indeed, in subsurface environments such as caves and mines, the *Proteobacteria* phylum is the second-most abundant, with e.g. *Coxiellaceae* identified in sediments from a cave in Thailand (1.5% of the microbial community) (Leon et al. 2018, Wiseschart et al. 2019, Bontemps et al. 2022). *Coxiella burnetii* was identified in a cave in Spain, where it caused a Q fever outbreak in 2021 (five cases) (Hurtado et al. 2023). Caves can be considered as a reservoir for pathogens, where *Legionella* are regularly identified (e.g. 3.3% in Bossea Cave, Italy) (Biagioli et al. 2023). The presence of *Legionella* is often associated with amoebae, which are generally present in caves. Indeed, the phylum *Amoebozoa* is often found in soil and water, a characteristic shared by karst environments (Alonso et al. 2019, Bontemps et al. 2022). Over nine species of *Legionella* associated with amoebae have been identified in Lascaux cave (among others *L. pneumophila, L. jordanis*, and *L. oakridgensis*) (Bastian et al. 2009). Like amoebae, host ticks of certain *Francisellaceae* and *Coxiellaceae* are found in subterranean environments with high humidity; this is the case for Agardsides and Carios ticks (Kazim et al. 2021).

The aerosols category represents only 2.1% of the samples positive for *Legionellaceae*, 0.3% and 0.1% for *Coxiellaceae* and *Piscirickettsiaceae*, respectively (Fig. 4B–D). The most common route of transmission of *Legionella*, in cases of legionellosis, is the inhalation of contaminated aerosols from contaminated water (Paschke et al. 2019). Sources of aerosols associated with this transmission include air conditioning cooling towers, hot and cold water systems, and so on. As a consequence, aerosols and cooling towers are frequently monitored for *Legionella-*related epidemiological reasons (Allegra et al. 2016, Paschke et al. 2019, Paranjape et al. 2020a), inherently increasing the number of samples found in this biome compared to other DIG families.

As expected, DIGs are most often found in aquatic environments, followed by animal guts and sediments, but also, increasingly, in saline environments (Fig. 4). This ecological description remains incomplete, with 25% of DIG-positive samples (up to 42% for the *Fastidiosibacteraceae*) having no associated biomes (Fig. 4).

### Environmental factors

**Figure 5.**
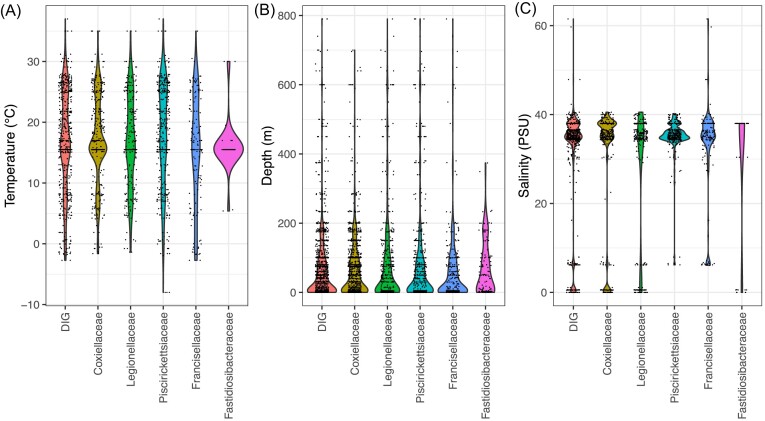
Distribution of DIGs across three main environmental factors: (A) temperature, (B) depth, and (C) salinity, shown for DIGs, *Coxiellaceae, Legionellaceae, Fastidiosibacteraceae, Francisellaceae*, and *Piscirickettsiaceae*.

It is increasingly recognized that the emergence, prevalence, and severity of diseases depend on complex interactions between pathogens, hosts, and the environment (Jones et al. 2008, Plowright et al. 2008). Several risk factors, both biotic (age and species of the host) and abiotic (temperature, salinity, sunlight, and hydrodynamic connectivity), are known to contribute to the occurrence of epidemics and the alteration of microbial diversity in natural environments. In this survey of the published metagenomic data, we recovered metadata for each metagenome available on MGnify (Gurbich et al. 2023), including depth, location, elevation, temperature, salinity, altitude, pH, latitude, longitude, nitrate, and oxygen concentration. However, metadata is often patchy, and no single metagenome has all this information. Here, we reviewed the effects of four environmental factors (temperature, depth, salinity, and pH) on the DIG community (Fig. 5).

Temperature is an important factor to consider, as rising global temperatures can lead to changes in the abundance of certain microorganisms and protists (Hosen et al. 2017). Despite the ease of measuring temperature, it is available only for a fraction of the samples, 28.7% of the data for *Francisellaceae*, 9.5% for *Coxiellaceae* and *Legionellaceae*, and 7.8% for *Fastidiosibacteraceae* (Fig. 5A). Within these, ~10% of the values are obviously erroneous (e.g. −9999.0°C or +9999.9°C), complicating downstream analysis, and highlighting the fact that too few users provide correct metadata information when submitting sequences. For DIGs in general, the average temperature at which they are identified is 16.9°C, with a range of −8°C to 37°C (Fig. 5A). It is known that e.g. *Legionella* can multiply at higher temperatures (e.g. Tison et al. 1980, Kusnetsov et al. 1996, Committee on Management of Legionella in Water Systems 2019). In Hot Springs National Park, Arkansas (USA), where hot spring water is piped at naturally high temperatures (>57°C), five cases of legionellosis occurred in 2018–2019, indicating the presence of *L. pneumophila* bacteria in piped spring water (James et al. 2022). Conversely, the presence of *L. pneumophila* has been reported in cold environments, such as in the freshwater lake of King George Island in the Antarctic Peninsula. Although the proportion of *Legionella* species is consistently low, while they have been frequently detected even in Antarctic lakes, showing their wide distribution in cold climates (Shimada et al. 2021). These results suggest that *Legionella* spp. adapted to extreme temperatures may be widely distributed in both low and high temperature environments. Temperature also affects the ability of DIGs to infect their hosts: for example, while at higher temperatures *L. pneumophila* is able to parasitize *A. castellanii* cells, below 25°C, *A. castellanii* has been observed to digest the *L. pneumophila* population infecting it (Moffat and Tompkins 1992).

In other DIG groups, temperature is a critical environmental factor in the development of outbreaks. A study in salmon farms in Chile, suggests that temperature is positively correlated with fish mortality due to piscirickettsiosis, caused by *Piscirickettsia* spp. The observed prevalence of piscirickettsiosis was around 20% when the water temperature was below 9°C, while it increased to 70% at temperatures above 9°C (Martínez et al. 2018, Bravo et al. 2020). Temperature is also a critical factor for francisellosis outbreaks, where water temperature below 26°C promotes the disease. A study conducted on *Francisella* shows that changes in temperature modulate the expression of oxidative stress and heat shock genes, as well as metabolism-related genes. The presence of DIG families over a wide range of temperatures (−8°C to 37°C) is also associated with the host being able to adapt more quickly to certain environmental conditions; however, temperature can affect both the host and its microbial partners (Lemoine et al. 2020). Indeed, thermal variations have a major impact on host metabolism, with extreme temperatures threatening survival and fertility. In addition, thermal stress can deplete obligate endosymbionts, destabilizing the association and reducing the fitness of both partners, thus limiting the host’s viable temperature range (Zhang et al. 2019). A rise in temperature has been shown to cause a modulation in the expression of genes involved in the innate and adaptive immune response in salmon and facilitate the development of *P. salmonis* (Martínez et al. 2018).

The most commonly reported metadata is the depth at which the samples were collected, with an average of 30.4% of the samples in our dataset containing this information, rising to 51.9% for *Francisellaceae* (Fig. 5B). No study has directly compared the effects of depth (i.e. the effects of different depths in the same ecosystems) on the abundance and diversity of DIGs. However, it can be assumed that depth is a factor that affects the structure of microbial communities, as it is associated with significant biotic and abiotic changes, such as the absence of light, CO_2_, oxygen, moisture, and nutrient availability (e.g. oligotrophy). For all the DIG families studied here, the depth data are similar and the average is around 0 m, meaning that most of the time they are identified when sampling at the surface, between the litter and mineral layer (Fig. 5B). Certain DIG families are found on the seafloor (e.g. deep-sea hydrothermal vents, see section above). Although most DIG families are found in aquatic environments, very little information is currently available on their presence at different levels of the water column.

Many studies have shown that salinity also alters the structure of the microbial community and reduces microbial activity. Salinity information is available for 11.6% of the DIG metagenomes, ranging from 0 to 61.3 practical salinity unit (PSU, gram of salt per kilogram of water) for *Fransicellaceae* (Fig. 5C). Indeed, a single *Francisella* species was detected by qPCR in waters ranging from 1 to 38 PSU (Brunet et al. 2021). The effect of salinity on the detection of *Francisella sp*. could not be evaluated in that study, due to the insufficient number of saline and brackish water samples. However, *Francisellaceae* are found in fresh, brackish, and saltwater, indicating that these bacteria can inhabit a wide range of water types (Fig 5C). Similarly, *Legionellaceae* have been identified in natural seawater from the Baltic Sea and the North Sea with salinities of 15–30 PSU (Linsak et al. 2021). Aquatic environments with low or moderate osmotic pressure are the main natural aquatic habitats for *Legionella* (Schwake et al. 2021). Although saltwater can cause environmental stress to bacterial cells, the natural presence of *Legionella* in this environment and their tolerance to it has long been reported. Research on *Legionella* ecology in saline springs is limited and often assumes freshwater contamination. Although this is often the case, reports from high osmolality, isolated, and oceanic sites suggest that saline environments may also provide a natural habitat for *Legionella* (Schwake et al. 2021). Exposure of *Legionella* to high concentrations of sodium has previously been shown to inhibit the growth and virulence of laboratory strains and promote the growth of avirulent forms (Bergman et al. 2023). The ability of *Legionella* to survive and multiply in their protozoan hosts is mediated by the T4BSS, which is also involved in the sodium sensitivity phenotype observed in *L. pneumophila*. It has been postulated that sodium sensitivity is a result of leakage of sodium ions through the T4BSS (Bergman et al. 2023). It is therefore likely that *Legionella* species have adapted to survive in sodium-rich environments, in the presence of sodium-resistant protozoa. Indeed, a previously unidentified species, *L. tunisiensis*, was isolated by coculturing amoebae in Lake Sabka, a hypersaline lake in Tunisia. Similarly the genus *Legionella* has been identified in wastewater, from the Great Salt Lake of Utah, USA, with salinities ranging from 3% to 140% (Schwake et al. 2021). Salinity also affects the prevalence of piscirickettsiosis, which is higher (74%) at salinities >26 PSU compared to a much lower prevalence (<29%) at salinities <26 PSU (Bravo et al. 2020). In addition to *P. salmonis*, several species of *Piscirickettsiaceae* have been isolated from marine, brackish, or lake habitats. This is the case, for example, of *F. adeliensis*, which is able to adapt to fluctuations in ambient salinity (0%–3.5%) (Vallesi et al. 2019).

Another key factor that determines the structure of a microbial community is pH. However, it is rarely reported: only 0.7% of the samples we investigated contained pH data. Variations in pH have a significant impact on the biological mechanisms of DIGs. For example, *C. burnetii*, the causative agent of Q fever, replicates in an intracellular phagolysosome at a pH between 4 and 5. Data show that *C. burnetii* is unable to grow in media with a pH of 6.0 or higher, but cells remain viable (Smith et al. 2019). Surprisingly, one study shows that *F. tularensis* subsp*. holarctica* grows at a reduced rate at pH 7.4 compared to pH 6.4, suggesting that pH 7.4 (i.e. the human body) is a challenge to which the bacteria must adapt (Wastella et al. 2018). Conversely, adaptation also occurs when *F. tularensis* is in a stressful condition with a low pH. Indeed, several changes in the pathogen’s machinery are observed, such as the cessation of maturation of vacuole-containing bacteria, the cessation of fusion with the lysosome and, on the contrary, a disintegration of the vacuole membrane by mechanisms not yet elucidated (Klimentova et al. 2019). Modification of pH can actually be used as an effective disinfectant in cooling towers: conditioning at pH ≥ 9.6 is considered an effective operation to control the pathogenicity of *L. pneumophila* (Pinel et al. 2021).

## Impact of molecular mechanisms on the mutualism–parasitism continuum of DIGs

**Figure 6.**
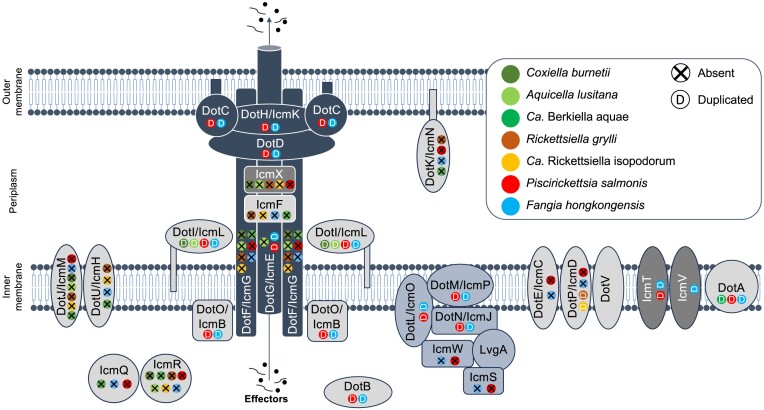
Protein composition of the *L. pneumophila* T4BSS. Differences with other DIGs T4BSS are highlighted with a cross when the protein is absent and with a ‘D’ when it is duplicated. Dark blue: core complex (DotC–DotD–DotH–DotF–DotG); light blue: core transmembrane subcomplex (DotL, DotM, DotN, IcmSW, and IcmS-LvgA); light grey: other functional proteins (DotF, DotE, DotV, DotP, DotI, DotJ, DotA, DotB, IcmQ, IcmR, IcmF, IcmH, DotK, and DotO); and dark grey: proteins with unknown functions (IcmT, IcmV, and IcmX). Based on Gomez-Valero et al. (2019) and Hugoson et al. (2022).

The host immune system is an important barrier against all colonizing microorganisms, from those that are harmful to those that are beneficial (Belkaid and Hand 2014). Mutualistic interactions with microbes have facilitated the radiation of the major eukaryotic lineages, e.g. mitochondria and chloroplasts (Wernegreen 2012). In particular, endosymbionts can provide biochemical capabilities such as photosynthesis, chemosynthesis, and nitrogen fixation that allow eukaryotes to adapt to new habitats or specialize in specific nutritional niches (Duron et al. 2018a). Conversely, parasitic interactions with pathogens have been facilitated by the deployment of virulence factors that allow them to subvert the host’s antimicrobial countermeasures. The more virulent a pathogen, the greater the degree of damage it can cause in the host, but virulence eventually evolves to a level that optimizes the reproduction and transmission rate of the pathogen (Gomez-Valero and Buchrieser 2019). Virulence has evolved through the coevolution of pathogens with their hosts, a major driver of evolution over millions of years. Host–pathogen coevolution is widespread in ecosystems, and resulted in the emergence of sophisticated mechanisms for disrupting host functions on one side, and shaping the immune defences of eukaryotic cells on the other side. In this section, we review two mechanisms that generate different kinds of interactions along the mutualistic–parasitic continuum between endosymbionts and their hosts. The first is the T4BSS (Fig. 6), which is found in most DIGs (all *Legionellales*, some *Francisellaceae*—but not *Francisella*—and *Piscirickettsia*) and is used by bacteria to inject proteins into the host cytoplasm to resist digestion during phagocytosis (Ghosal et al. 2019). The second is the synthesis of biotin (also known as vitamin H or B7), an essential cofactor that is synthesized by a variety of conserved pathways and may be required for the virulence of certain pathogenic bacteria (Sirithanakorn and Cronan 2021). These different systems will be reviewed, highlighting their conservation status and their role within DIGs.

### The Dot/Icm T4BSS

Type IV secretion systems (T4SS) are nanomachines produced by Gram-negative and Gram-positive bacteria to transport macromolecules across their membranes. The systems are used for a wide range of functions, including the exchange of genetic material between bacterial species, the acquisition of novel genetic material from the external environment, and the delivery of nucleoprotein complexes or effector proteins to recipient cells (Wallden et al. 2010). Several T4SS are present in DIGs, but one stands out as critical for their interaction with their host: the T4BSS, which translocates proteins to the host cytoplasm. It consists of one or several operons coding for proteins called intracellular multiplication (Icm) or defects in organelle trafficking (Dot) (Vogel et al. 1998, Segal et al. 2005). The structure and function of the proteins constituting the T4BSS in *L. pneumophila* have been extensively reviewed recently (e.g. Lockwood et al. 2022), and will only be briefly summarized here. This T4BSS, also referred to as Dot/Icm system consists of about 27 proteins, including an outer membrane protein (DotH), outer membrane lipoproteins (DotC, DotD, and DotK), a periplasmic protein (IcmX), inner membrane proteins (DotA, DotE, DotF, DotG, DotI, DotJ, DotM, DotP, DotU, DotV, IcmF, IcmT, and IcmV), inner membrane-associated ATPases (DotB, DotL, and DotO), and soluble cytosolic proteins (DotN, IcmQ, IcmR, IcmS, and IcmW) (Fig. 6) (Ghosal et al. 2019). The T4BSS is usually subdivided into a core transmembrane subcomplex (mainly DotC, DotD, DotF, DotG, and DotH) and a coupling protein subcomplex (mainly DotL, DotM, DotN, IcmS, IcmW, and LvgA) (Ghosal et al. 2019). The T4BSS plays a key role in the interaction of *Coxiella* (van Schaik et al. 2013), *Rickettsiella* (Bouchon et al. 2011), and *Legionella* (Segal et al. 2005, O’Connor et al. 2011) with their hosts, by injecting a variety of protein effectors (see section below) into the host cell. Within the host cell, Dot/Icm substrates have been shown to manipulate various host cellular processes, enabling the establishment of a replication vacuole and intracellular growth in *L. pneumophila* and *C. burnetii* (Kidane et al. 2022). More recently, the discovery of T4BSS homologs in members of the *Francisellaceae* and *Piscirickettsiaceae* pushes the origin of T4BSS back to the common ancestor of the orders *Legionellales* and the two above-mentioned families. For example, a Dot/Icm system has been identified in the fish pathogen *P. salmonis*, where the phagosome–lysosome fusion event is inhibited during *Piscirickettsia* infection (Mauel et al. 1999, Fa et al. 2013). A homologous T4BSS is also present in *F. hongkongensis* (*Fastidiosibacteraceae*), possibly acquired by horizontal gene transfer (HGT) from *Piscirickettsia* (Hugoson et al. 2022).

T4BSS is found in all *Legionella* species studied (except the very reduced insect endosymbiont ‘*Ca*. Legionella polyplacis’), in *C. burnetii*, the etiological agent of Q fever, in *P. salmonis* and in *Rickettsiella grylli*, and is essential for their intracellular growth (Hugoson et al. 2022). The functional similarities between the T4BSS of *C. burnetii* and *L. pneumophila* are well known. For example, the *C. burnetii* Nine Mile RSA439 genome encodes homologs of 23 of the 26* L. pneumophila* Dot/Icm proteins, with amino acid sequence identities ranging from 22% to 66% (Larson et al. 2023). In the genomes of ‘*Ca*. Berkiella cookevillensis’ and ‘*Ca*. Berkiella aquae’, 17 and 18 of the 26 Dot/Icm genes share a high degree of homology with those of *L. pneumophila* (Kidane et al. 2022). The situation is similar in *P. salmonis*, where almost all T4BSS proteins described in *L. pneumophila* and *C. burnetii* have been identified in the *P. salmonis* proteome, including core proteins DotH, DotG, DotF, and DotD (Cortés et al. 2017). However, the T4BSS of these four genera (i.e. *Legionella, Coxiella, Rickettsiella*, and *Piscirickettsia*) show differences (Burstein et al. 2016, Grohmann et al. 2017). Indeed, while IcmQ orthologs are found in the *Legionella, Coxiella*, and *Rickettsiella* Dot/Icm systems, there is a lack of interspecific complementation by IcmQ for the latter two genera (Segal et al. 2005). This is expected, as the *L. pneumophila* IcmQ protein interacts with the IcmR protein, which is absent from *Coxiella* and *Rickettsiella*, even though functional IcmR homologs have been found in both organisms (Nagai and Kubori 2011, Grohmann et al. 2017).

The *C. burnetii* genome also lacks DotJ, but has a tandem duplication of the gene coding for DotI, while *R. grylli* has both DotI and DotJ proteins (Segal et al. 2005, Nagai and Kubori 2011). DotJ/IcmM and DotI/IcmL genes in *L. pneumophila* are homologous to each other. DotI and DotJ are tightly bound, inner membrane integral proteins that are essential for Dot/Icm-dependent activities. DotJ consists only of the conserved N-terminal region, whereas DotI has an additional periplasmic domain (Ghosal et al. 2019). The similarity between DotJ and DotI suggests that the DotI duplication can substitute for the absence of DotJ, the functional homolog of IcmR (Fig. 6) (van Schaik et al. 2013). This suggests that the DotI gene duplication occurred in a common ancestor of *Legionella, Coxiella*, and *Rickettsiella*, and that the DotJ-like protein evolved after species differentiation.

DotV, IcmF, and DotU/IcmH are also absent in *Rickettsiella*, while IcmF is fragmented in *Coxiella* (van Schaik et al. 2013, Christie et al. 2017). The two genes located at the 5' end of the Dot/Icm II region in *L. pneumophila* (*icmHF*) were found to be 1270 kb away from the rest of the Dot/Icm genes in *C. burnetii*, and one of these genes (*icmF*) was interrupted by an early stop codon.

A phylogenetic study of *Rickettsiella popilliae* and *R. grylli* showed that these bacteria carry genes orthologous to *dotB* and *dotO* (Nagai and Kubori 2011). An interspecific complementation study of *L. pneumophila* Dot/Icm mutants with homologous *C. burnetii* Dot/Icm proteins was performed. This analysis identified four *C. burnetii* Dot/Icm proteins that can be expected to replace their *L. pneumophila* homologues (IcmS, IcmW, IcmT, and DotB) and six other proteins (IcmQ, IcmP/DotM, IcmO/DotL, IcmJ/DotN, IcmB/DotO, and IcmX) that cannot (Zamboni et al. 2003, Segal et al. 2005, van Schaik et al. 2013). This functional substitution of their *L. pneumophila* counterparts in intracellular replication showed that the *Coxiella* Dot/Icm system is functional and plays an essential role in interactions with its host cells (Zamboni et al. 2003, Zusman et al. 2003).

Differential expression of *dotH* and *dotG* was observed in *P. salmonis*, which may correlate with the establishment of the infectious process when bacteria inject virulence factors into host cells. The secretion of effector proteins by the T4BSS in *P. salmonis* could promote lysosomal escape of bacteria and favour intracellular survival. Unlike bacteria such as *Legionella* or *Coxiella*, which have multiple divergent copies of T4BSS genes, reflecting functional diversity, *P. salmonis*, shows divergence in the expression levels of T4BSS genes rather than in copy number (Fig. 6) (Nourdin-Galindo et al. 2017).

Studies have shown that the T4BSS of the genus *Legionella* harbours three *Legionella-*specific proteins, IcmX, DotJ, and DotF. The first is subject to high recombination rates (Gomez-Valero and Buchrieser 2019), located on the surface of the bacteria, and therefore likely to be exposed to the host. The second, DotJ, is located in the inner membrane and is a distant paralog of DotI, with which it forms a heteroduplex (Kuroda et al. 2015). The third, DotF, has a periplasmic portion that would be under positive selection and would be involved in substrate recognition (Sutherland et al. 2013).

Even if the Dot/Icm system is part of the core *Legionella* genome, some differences in the organization of the constituent proteins are observed between the different *Legionella* species, even if it remains highly conserved (Christie et al. 2017, Gomez-Valero and Buchrieser 2019). Indeed, these differences are due to gene insertions of variable sizes, for example, a seven-gene insertion between *icmB* and *icmF* in *L. brunensi*, compared to a single gene between the same two genes in *L. pneumophila*, and no intermediate genes in *L. quinlivanii* (Burstein et al. 2016). While these inserted genes have not been definitively linked to the Dot/Icm secretion system, the possibility that they might play a role in this system, potentially acquired through HGT, should not be discounted.

### Diversity and functions of the effectors

Intracellular growth of DIGs requires the T4BSS, encoded by the *d ot*/*icm* genes (see section above), which translocates bacterial proteins into the host cytoplasm, where they manipulate various host cellular processes (O’Connor et al. 2011). These proteins, called effectors, play a major role in DIG virulence and are well-described in *L. pneumophila*, the causative agent of Legionnaires’ disease. The function of these effectors has also been recently reviewed (e.g. Lockwood et al. 2022, Chauhan et al. 2023). Effectors modify the host behaviour in multiple ways, but their effects can be broadly divided in two categories. The first is to avoid being killed by the host, among others by preventing the fusion of the phagosome with the lysosome, by modulating signalling pathways involved in pathogen recognition, or by preventing apoptosis. The second is to favour the exploitation of the host’s resources by manipulating the endosome trafficking system and hijacking the ubiquitin pathway.

In *Legionella*, effectors make up about 10% of the genome (Grohmann et al. 2017, Hilbi et al. 2017). Studies show that the pangenome of the genus *Legionella* contains over 1600 families (18 000 genes) of protein effectors, but that only eight are conserved across the genus (Burstein et al. 2016, Hilbi et al. 2017, Gomez-Valero et al. 2019). Only a subset of these effectors have been characterized, and in many cases mutations of the effector genes do not lead to discernible phenotypes, probably due to functional redundancy. To date, some of the effectors have been shown to play a role in modifying host cellular processes to establish a connection between the bacteria and the host cell, allowing *L. pneumophila* to colonize the host cell (Kubori and Nagai 2016). While the T4BSS is highly conserved, most effectors are dispensable when deleted individually. Indeed, simultaneous deletion of multiple substrates had little effect on mouse macrophage growth (O’Connor et al. 2011). However, the repertoire of effectors varies considerably between these species. An analysis of putative T4BSS effectors from *Legionella longbeachae*, the second most common cause of Legionnaires’ disease, revealed that only about 50% of the virulence factors described in *L. pneumophila* were also present in the *L. longbeachae* genome (Gomez-Valero et al. 2019). One study found that 258 effectors (42%) were species-specific, i.e. observed in only one of the 50 *Legionella* species analysed (Burstein et al. 2016). Excluding *L. pneumophila*, the species with the highest number of unique putative effectors is *L. waltersii*, with 23 specific effectors. In addition, *L. pneumophila* encodes a unique family of translocated effectors called meta-effectors, which are effectors that interact with each other and regulate other *L. pneumophila* effectors in host cells (Urbanus et al. 2016). A genetic screen identified 20 possible interactions between effectors. However, for most meta-effectors, no direct interactions have been experimentally demonstrated. Currently, 10% of *L. pneumophila* effectors have been shown to be part of the effector protein interactome, suggesting a strong presence of meta-effectors (Grohmann et al. 2017). One study has recently described the meta-effector, MesI, which promotes *L. pneumophila* virulence by regulating the cytotoxic effector SidI. When MesI and SidI are uncoupled, SidI is toxic to *L. pneumophila* (*in vitro*) and induces severe bacterial degradation in host cells. Furthermore, MesI translocation appeared to be essential for intracellular replication, demonstrating that intrabacterial regulation of SidI contributes to *L. pneumophila* virulence (van Schaik et al. 2013, Chauhan et al. 2023). Meta-effectors are a key element of *L. pneumophila* virulence, as several are required for intracellular replication. Recently, para-effectors (from the Greek para meaning *besides* but also *contrary to*), a new category of effectors has been introduced (Schator et al. 2023). Some effectors function as pairs of highly interdependent effectors that fine-tune host cell gene expression and promote bacterial intracellular replication. This has been demonstrated for two chromatin modifying effectors (RomA and LphD) in *L. pneumophila*. Using mutation and virulence assays, it was shown that the presence of only one of these two effectors impairs intracellular replication, whereas a double knock-out (*∆lphD ∆romA*) can restore intracellular replication (Schator et al. 2023).

The effectors that pass through the T4BSS of *C. burnetii* have not been the subject of such in-depth studies, largely due to the difficulty to grow *Coxiella* axenically (Beare et al. 2009). To date, 150 protein substrates have been identified in *C. burnetii*, representing ~6% of the open reading frames in its genome (Chen et al. 2010, Carey et al. 2011, Qiu and Luo 2017). Only a few *C. burnetii* effector proteins have been functionally characterized, with roles in interfering with vesicular trafficking, host transcription, and apoptosis (Larson et al. 2016, 2019, Lührmann et al. 2017). In some cases, the cellular targets of the effectors have been identified. For example, AnkG, CaeA, and CaeB can inhibit host cell apoptosis. More specifically, CaeA localizes to the nuclei of infected cells where it activates the expression of surviving, an inhibitor of activated caspases, which can contribute to the induction of apoptosis (van Schaik et al. 2013, Qiu and Luo 2017). Other effectors are specifically involved in lipid metabolism of the *Coxiella*-containing vacuole, host gene expression, autophagy, cell death, and immunity (Qiu and Luo 2017, Larson et al. 2023). Only six *C. burnetii* effectors have homologs in *L. pneumophila*, and in contrast to the extensive functional redundancy between *L. pneumophila* effectors, *C. burnetii* mutants lacking a single effector often lose the ability to grow optimally in host cells (Hilbi et al. 2017, Lührmann et al. 2017). The apparent lack of redundancy between effectors in this species may be due to the comparatively narrow host range of *C. burnetii* compared to *L. pneumophila*, suggesting their important role in virulence and host adaptation. (Duron et al. 2018a, Santos-Garcia et al. 2023).

Only four effectors have been identified by genetic screening in *P. salmonis*, all homologous to *L. pneumophila* and *C. burnetii* effectors (Grohmann et al. 2017). The four proteins have a typical Dot/Icm secretion signal at the C-terminus and are overexpressed during *in vitro* infections, suggesting a potential involvement in intracellular survival and proliferation of *P. salmonis* (Labra et al. 2016). Furthermore, these four effectors all have eukaryotic-like protein domains, revealing a possible common function during translocation to the eukaryotic host during the establishment of pathogen–host relationships.

Effectors may also play a role in environmental adaptation, as suggested by an analysis that revealed 51 new putative effectors specific to *L. longbeachae*, which may be related to its different lifestyle compared to *L. pneumophila* (Gomez-Valero et al. 2019). Indeed, *L. longbeachae* is found in wet soils and loams, and genes possibly originating from plants have been identified, such as proteins with pentatricopeptide repeat domains, a significantly protein family in plants. A subpopulation of effectors has also been correlated with host-specific replication of *L. pneumophila* in *A. castellanii, A. polyphaga, H. veriformis*, and *N. gruberi* (Chauhan and Shames 2021). The central effector MavN, which plays a role in environmental adaptation, has also been identified in *L. pneumophila* and *R. grylli* (Burstein et al. 2016). MavN was recently found to be an iron transporter (Christenson et al. 2019).

The evolution of multiple mechanisms to adapt to different environmental conditions and subvert the eukaryotic host is likely due to the molecular arms race that has developed between different DIG organisms and the broad spectrum of protozoan hosts encountered in their natural environment (Grohmann et al. 2017). This suggests that many effectors with potentially important functions in virulence have recently been acquired by HGT, highlighting DIGs as a highly dynamic system capable of rapid adaptation (Burstein et al. 2016, Říhová et al. 2017).

### Eukaryotic domains

Effector proteins often carry conserved eukaryotic protein domains. A eukaryotic domain has been defined as a protein domain found in >75% of eukaryotic genomes and <25% of prokaryotic genomes (Mondino et al. 2020). These eukaryotic protein domains are very important in host–microorganism interactions: once transferred into the microorganism genome, their original function that was in the host is altered to the benefit of the microorganism. Indeed, the majority of these genes are likely to have been acquired by HGT directly from protozoa, highlighting the importance and coevolution of DIG interactions with their host (Chauhan and Shames 2021)

The most abundant eukaryotic domains identified in DIGs are ankyrin repeats (Gomez-Valero and Buchrieser 2019). Indeed, more than 20 genes encoding proteins containing eukaryotic ankyrin repeats have been identified in *Coxiella*. Ankyrin repeats are protein–protein interaction motifs of tandemly repeated modules of 30–34 amino acids found in eukaryotic proteins involved in various cellular functions, including transcriptional regulation, signal transduction, vesicular trafficking, and cytoskeletal integrity (Kidane et al. 2022). A large majority of the effectors identified in the genomes of *P. salmonis, Legionella santicrucis*, and *Legionella massiliensis* harbour ankyrin repeats, with the latter two genomes encoding 41 and 39 ankyrin domains, respectively. Ankyrin motifs are frequently associated with other eukaryotic motifs and are associated with eukaryotic F-box, U-box, Rab, or SET domains, suggesting that manipulation of the host ubiquitin system is a fundamental virulence strategy (Labra et al. 2016, Grohmann et al. 2017, Qiu and Luo 2017, Gomez-Valero et al. 2019, Chauhan and Shames 2021, Kidane et al. 2022). F-box-like domains are also well described in *L. pneumophila* and *C. burnetii*. Based on their functions in eukaryotic cells, this group of proteins presumably promotes proteasome-mediated protein degradation (van Schaik et al. 2013). For example, AnkB (F-box protein) in *L. pneumophila* targets nonessential host proteins (via ubiquitylation) for degradation by the 26S proteasome, thereby providing an energy source for its growth. *L. pneumophila* also mimics eukaryotic RNAs, such as miRNAs, to interfere with eukaryotic regulatory mechanisms. For example, *L. pneumophila* uses extracellular vesicles to translocate small bacterial RNAs (e.g. RsmY and tRNA-Phe) into host cells to interfere with host defence pathways (i.e. downregulating the expression of key sensors and regulators of the host cell innate immune response) (Sahr et al. 2022).

### Biotin

Biotin synthesis is also essential for the virulence of some human pathogens, such as *F. tularensis* and some *Coxiellaceae* (Santos et al. 2003, Feng et al. 2014). Biotin (vitamin B7 or H) is essential in all aspects of life as it plays a key role in central metabolic processes involving carboxylation, decarboxylation, and transcarboxylation (Sirithanakorn and Cronan 2021). The function of biotin synthesis is well described for *Francisella*. A *Francisella* protein, FTN_0818, was identified as involved in biotin biosynthesis and at the same time essential for intracellular replication. It is required for rapid escape from the *Francisella*-containing phagosome, suggesting that biotin may be a limiting factor: when absent, it confines cytosolic pathogens to the phagosome, blocking their escape and preventing them from reaching their replication niche in the cytoplasm. Biotin sequestration may be a form of nutritional immunity by the host’s innate immune system and supports the idea that biotin may be a critical and limited resource during infection and is therefore correlated with *Francisella* virulence (Napier et al. 2012, Feng et al. 2014, Duron et al. 2018b). The role of biotin synthesis in DIG virulence has also been highlighted in *C. burnetii*. Indeed, blocking biotin biosynthesis inhibits the growth of *C. burnetii* on specific axenic media, suggesting that biotin is required for the growth and development of this pathogen (Moses et al. 2017, Duron et al. 2018b, Santos-Garcia et al. 2023). However, the biochemical/metabolic mechanism used by some DIGs to synthesize biotin and thus facilitate their infectivity remains unknown. On the other end of the spectrum, the acquisition—through HGT—of a biotin synthesis operon contributed to shifting the position of a *Legionella* species along the parasitism–mutualism continuum. Indeed, in contrast to other *Legionella* species, which are facultative intracellular pathogens of various protists, ‘*Ca*. Legionella polyplacis’, is an obligate mutualistic endosymbiont of ticks, presumably providing its host with biotin (Říhová et al. 2017).

In summary, the diverse repertoire of effectors in different DIG species reflects a high degree of evolutionary plasticity, with constant acquisitions, losses, and adaptation of genetic material from host cells or coinfecting pathogens supporting DIG virulence. However, the inability to identify effectors remains a major challenge in the study of host–DIG interactions along the mutualism–parasitism continuum. Furthermore, a better characterization of effectors will allow us to know whether the effector repertoire of a particular DIG strain reflects the ecosystem from which it was originally isolated, and whether the hosts of that particular niche have an impact on the composition and diversity of effectors.

## Influence of the microbiota—interaction with other bacteria and endosymbionts

Most ecosystems are populated by large numbers of diverse microorganisms that interact with each other to form complex networks of ecological interactions. The possible combination of positive (+), negative (−), or neutral (0) outcomes for both partners in the interaction makes it possible to classify different types of interactions along the mutualism–parasitism continuum, for example mutualism (+,+), commensalism (0,+ or +,0), amensalism (0,− or −,0), parasitism (+,− or −,+), and competition (−,−) (Lidicker 1979, Faust and Raes 2012). Some of these microorganisms can colonize the surface and/or internal parts of other organisms such as plants and animals, adding a further level of complexity to the interactions. In addition, abiotic factors (e.g. physical and chemical parameters) also shape the nature and complexity of microbial interactions (Paniagua et al. 2020). These microbial interactions can be both intraspecific and interspecific, ranging from simple short-term interactions to complex long-term interactions (Moënne-Loccoz et al. 2014).

All DIGs are present in a wide range of ecosystems (see above, section - Environmental distribution of DIGs) and interact along the mutualism–parasitism continuum with various microorganisms present in the community. Due to their distribution and lifestyle (i.e. free-living and intracellular), the nature and underlying mechanisms of these interactions are often unknown and difficult to study within ecosystems. In this section, we will review the interactions between DIGs and different microorganisms in three different contexts: interactions outside the host, intracellular interactions, and interactions within multicellular hosts.

### Interactions outside the host

Despite their mainly intracellular lifestyle, DIGs have been observed to interact with different microorganisms in biofilms when they are outside of their host. Biofilms represent important environments for the ecology of several DIG species, as they provide several functions, such as protection from biocides, a source of nutrition, and a source of dissemination (Stewart 1993, Kim et al. 2002, Temmerman et al. 2006, Hsu et al. 2011). Moreover, as biofilms contain the bulk of microbial biomass, they attract microbial grazers and are where most biological interactions occur between microbial species (Flemming et al. 2002, Lau and Ashbolt 2009).

Protozoa are microbial grazers that play a significant role in shaping and structuring the bacterial community of an ecosystem, particularly in biofilms: they impact the microbial loop, carbon flux, and nutrient cycling of biofilms (Hahn and Höfle 2001, Bonkowski 2004). DIGs that infect and kill protozoa will reduce the level of grazing and reduce the pressure on the heterotrophic and primary producer populations in an ecosystem. This may explain the establishment of positive interactions between DIGs and other microorganisms in the community. For instance, *Flavobacterium breve, Brevundimonas* sp., and *Fischerella* sp. (cyanobacteria isolated from microbial mats), have been observed to nutritionally supplement *L. pneumophila* strains (Tison et al. 1980, Wadowsky and Yee 1983, Paranjape et al. 2020a). Though the exact nature and reason for this supplementation are unknown, it is thought to allow increased numbers of *L. pneumophila* around the supplier species. This in turn increases the probability that *L. pneumophila* cells are ingested by grazers and decreases the chances of the supplier species being ingested (Paranjape et al. 2020b). An added benefit is that grazers may eventually die if they are sensitive to *L. pneumophila*. Conversely, other species in the biofilm community can negatively interact (i.e. amensalism, parasitism, and competition) with some DIG species. Thus, species in a biofilm can produce antimicrobial molecules, which can either inhibit or kill DIG species. For instance, *Bacillus, Pseudomonas, Stenotrophomonas, Chryseobacterium, Staphylococcus, Cupriavidus, Aeromonas*, and *Flavobacterium* can inhibit *L. pneumophila* through the production of various antimicrobials (e.g. proteases and surfactin), leading to the establishment of amensalism interactions (Loiseau et al. 2015, Faucher et al. 2022, Paranjape et al. 2022). As DIGs contain several human pathogens, identifying and isolating species that can produce antimicrobials effective against these pathogens may prove valuable for public health issues.

Some eukaryotic and bacterial species in biofilms can also physically damage or destroy DIGs, and are usually predators or parasites. For instance, the eukaryotic heterolobosean amoeba *Solumistris palustris* and several cercozoan morphotypes consume *L. pneumophila* cells for nutrition (Anderson et al. 2011); in contrast, *L. steelei* is able to destroy *S. palustris* through a food poisoning-like method, indicating a DIG’s specificity in the predator–prey relation (Amaro et al. 2015). Another example of predatory interaction is the bacterial species *Bdellovibrio bacteriovorus*, which has been shown to prey on several species of *Legionella* and *Francisella* (Tomov et al. 1982, Russo et al. 2015). Finally, bacteriophages can be considered as antagonists, but research on phage–DIG relationships is still limited and controversial (see *L. pneumophila* phage in Cavallaro et al. 2022). One potential hurdle for the study of these phages may be the requirement of a tripartite model involving a phage, a DIG species, and an appropriate host species. Nonetheless, several viral genetic elements have been discovered in various DIG species, such as complete prophage sequences in *Legionella micdadei, Cysteiniphilum littorale* (*Fastidiosibacteraceae*), and *Francisella hispaniensis*, and CRISPR–Cas systems in *L. pneumophila* and *F. novicida* (Schunder et al. 2013, Gomez-Valero et al. 2014, Tlapák et al. 2018, Deecker et al. 2021, Qian et al. 2023). Furthermore, viral particles have been observed and imaged to infect *L. pneumophila* and *P. salmonis*, and recently UV radiation was shown to induce the assembly of noninfectious viral particles in *F. hispaniensis* (Yuksel et al. 2001, Lammertyn et al. 2008, Köppen et al. 2021). All in all, these results suggest that phages do exist and interact negatively with DIGs in nature, but more research is needed to confirm this hypothesis.

Indirect interactions between DIGs and several species also take place in the biofilm environment. An indirect biological interaction can be defined as the influence that an organism has on another through its interaction with another organism (Moon et al. 2010). A classic example is the trophic cascade effect where predation of one organism either positively or negatively affects another organism (Menge 1995). The parasitism of protozoan grazers by DIGs, such as *L. pneumophila* already mentioned above, illustrates the indirect effect of a trophic cascade on the grazer’s prey population. The interactions between *L. pneumophila*, amoebae and nematodes have been documented in biofilms and are examples of trophic cascade (Brassinga et al. 2010, Rasch et al. 2016). For example, the nematode *Plectus similis* feeds on various bacteria and amoebae, such as *A. castellanii*. The nematode, when presented with free-living *L. pneumophila*, displays reduced feeding behaviour than when presented with *Escherichia coli*, suggesting that it actively avoids ingesting the former. (Hemmerling et al. 2023). However, the nematode will feed on *A. castellanii* infected with *L. pneumophila* at similar rates than noninfected *A. castellanii*, allowing colonization of the gut of the nematode by *L. pneumophila*, which would not happen if the bacteria was free living (Hemmerling et al. 2023). The impact of the colonization of *L. pneumophila* on the gut of the nematode is not well-understood yet, but there is some emerging evidence that *L. pneumophila* negatively impacts the nematode *C. elegans* (Rasch et al. 2016), which is corroborated by the fact that free-living *L. pneumophila* are not preferred by *P. similis*.

Probiotic approaches have garnered interest as a potential method to reduce or eliminate pathogens, such as *L. pneumophila*, from various relevant ecosystems, including human gut or engineered water systems (Wang et al. 2013, Veiga et al. 2020, Cavallaro et al. 2022). However, with these approaches, indirect biological interactions have to be taken into account. Indeed, the purposeful elimination from an ecosystem of *L. pneumophila*, known to dominate water systems over other *Legionella* species, for example by adding the predator *S. palustris*, could increase the abundance of non-*pneumophila Legionella* species, which might in turn also be pathogens for humans (Wéry et al. 2008, Amaro et al. 2015, Salinas et al. 2021). Thus, the study of the indirect biological interactions of the members of the microbiome will undoubtedly be useful to predict, verify, model, and assess control strategies of pathogens.

### Intracellular interactions

Many DIG species are associated with environmental unicellular protozoans, and the biological interactions that occur between these two actors are numerous and complex. Protozoan hosts are a source of nutrition for DIG species that are colonized by them. Nutrition is usually acquired through parasitic interactions, with species within the *Legionella* genus being a prime example (Isberg et al. 2009, Gomez-Valero and Buchrieser 2019). Mutualistic nutritional interactions have yet to be observed between DIGs and protozoa; however, novel species of DIGs reside in amoebas without causing lysis. ‘*Ca*. Rhogoubacter’, ‘*Ca*. Pokemonas’, ‘*Ca*. Fiscibacter’, ‘*Ca*. Occultobacter’, and ‘*Ca*. Nucleophilum’ are all candidate genera closely related to the *Coxiella* and *Legionella* genera (Schulz et al. 2015, Pohl et al. 2021, Solbach et al. 2021). In these organisms, the lack of lysis of the host upon infection suggests a mutualistic relation between the partners, potentially based on nutritional supplementation. Moreover, a recently discovered and very reduced endosymbiont (‘*Ca*. Azoamicus ciliaticola’) displays a clear mutualistic relationship with its host, an anaerobic, amitochondriate ciliate, where the mitochondrial energetic functions have been replaced by the DIG endosymbiont (Graf et al. 2021).

Protozoan host species are also a source of protection for DIG species. Several protozoan species are known to encyst, providing protection from environmental stresses for bacteria—including DIGs, in particular *Legionella* spp.—present within them (Lambrecht et al. 2015). As bacteria can avoid detection when localized in cysts, this phenomenon has qualified protozoa hosts as ‘Trojan horses’ (Barker and Brown 1994). In a different example of protection, *Caedibacter taeniospiralis* (*Fastidiosibacteraceae*), a mutualistic endosymbiont of *Paramecium* species, is capable of conferring a killer trait to its host by the production of R-bodies, constituted of insoluble protein ribbons (Heruth et al. 1994, Beier et al. 2002). *Paramecium* cells that do not harbour the endosymbiont and ingest the R-bodies die from a toxin-related mechanism (Schrallhammer et al. 2018). The *Paramecium*-containing endosymbionts thus gain an advantage for resources and protection by destroying their competitors. Another function of protozoan hosts, and especially free-living amoebae, that has been hypothesized is that they serve as ‘training grounds’ for the development of intracellular bacterial human pathogens (Molmeret et al. 2005). This hypothesis is based on the fact that grazing protozoa place selection pressure on their prey and generate resistance to grazing (Amaro et al. 2015). This resistance can then be applied to human immune cells, such as macrophages, that use phagocytosis to kill pathogens. For example, the *Legionella* and *Francisella* genera contain species that are pathogenic to humans and acquired their virulence factors from their coevolution with amoebae (Barker and Brown 1994). Interactions between protozoa and DIGs can lead to the emergence of several DIG species with negative consequences for humans.

As well as interacting directly with their unicellular host, DIGs can also interact with its microbiota. In fact, unicellular protozoa can contain small microbiota, ranging from one mutualistic endosymbiont to several dozen genera of different bacteria (Delafont et al. 2013, Tsao et al. 2017, Solbach et al. 2021, Delumeau et al. 2023). Some social amoebae, such as *Dictyostelium discoideum*, can ‘farm’ their microbiome for their own benefit (Brock et al. 2011). One function for this microbiota is to defend the host against parasitic DIGs of amoeba. For example, *Acanthamoeba* S13WT harbours the chlamydial endosymbionts *Protochlamydia amoebophila* and *Neochlamydia* sp. These two endosymbionts protect their host against infections of *L. pneumophila* (Maita et al. 2018, König et al. 2019). The protection mechanisms are not fully understood, and it has been suggested that they are different for the two endosymbionts. For *P. amoebophila*, the endosymbiont changes the expression profile of the infecting *L. pneumophila* so that it cannot progress to its transmissive phase, making it impossible to infect subsequent amoeba hosts in the culture (König et al. 2019). In contrast, *Neochlamydia* is suggested to prevent *L. pneumophila* from entering the host (Maita et al. 2018). Moreover, coinfection studies of macrophages suggest that DIGs can either have neutral or beneficial interactions with other microbes in the intracellular medium. Indeed, coinfection of *C. burnetii* and *L. pneumophila* in various immune cells showed that both DIGs were compartmentalized in different vacuoles (Sauer et al. 2005). This compartmentalization appeared to separate both DIG species and limit competition, creating a neutral effect on the two pathogens’ ability to grow inside the immune cells. Moreover, coinfection between *L. pneumophila* and *Brucella neotomae* in macrophages suggested that *L. pneumophila* promotes the growth of the latter bacterial species (Kang and Kirby 2017). This interaction was parasitical as *B. neotomae* reduced the fitness of *L. pneumophila* (Kang and Kirby 2017). In another example, different species of amoebae infected with *L. pneumophila* were found to ingest fewer *E. coli* cells (Shaheen and Ashbolt 2021).

### Interaction inside multicellular hosts

A large number of DIG species form relationships with arthropods. They can be found in salivary glands, in the midgut, or malpighian tubules, (Braendle et al. 2003, Klyachko et al. 2007, Duron et al. 2020); in contrast to other symbiont groups, DIGs are often not located in specialized organs or cells. Some DIG species have established mutualistic interactions with arthropods, such as *Coxiella*-like endosymbionts present in oocytes in ticks (through vertical transmission) (Baumann 2005, Klyachko et al. 2007) and *Francisella*-like endosymbionts, which dominate in the microbiome of American dog ticks (Travanty et al. 2019). The most widely described mutualistic interaction is through vitamin B supplementation provided by the endosymbiont to its host. For instance, a *Coxiella*-like endosymbiont of Lone star ticks provides vitamin thiamine (B1), riboflavin (B2), niacin (B3), pantothenic acid (B5), pyridoxine (B6), and folate (B9) to its host; similarly, a *Francisella*-like endosymbiont of hard ticks harbours the complete pathway for vitamins B2, B7 (biotin), and B9 (Smith et al. 2015, Gerhart et al. 2018). Vitamin B supplementation seems to be a convergent adaptation to the nutritional needs of the bloodsucking host as blood meals are deficient in vitamin B (Duron et al. 2018b). Therefore, this supplementation by mutualistic DIGs can be observed for various bloodsucking arthropods, such as lice and ticks (Smith et al. 2015, Říhová et al. 2017, Duron et al. 2018b). Genes related to vitamin B metabolism have been acquired horizontally by these DIGs, suggesting a relatively recent endosymbiosis event (Smith et al. 2015, Říhová et al. 2017). To date, vitamin B supplementation is the only known mutualistic interaction between arthropods and DIGs, probably because most of the research has been focused on bloodsucking arthropods.

Though vitamin supplementation seems to be the most prevalent interaction between DIGs and arthropods, the dynamics of these interactions are quite diverse. Indeed, certain symbioses seem to occur in a duality with two species of bacteria that complement each other’s metabolic pathway for the nutrient required for the host. This has been observed in the *Hyalomma marginatum* tick, where a *Francisella*-like endosymbiont supplements the host with folate and riboflavin. This endosymbiont, however, lacks the capacity to produce biotin, due to extensive genome degradation (Buysse et al. 2021). Compensation for biotin occurs through *Midichloria* species, which have intact genes for biotin synthesis (Buysse et al. 2021). In contrast, certain DIGs such as *Coxiella*-like, *Francisella*-like, and *Rickettsiella* have established competition-type interaction in various tick species. Several of these species appear to offer the same type of nutritional supplementation interaction with their host. In this case, there is a competition between the various species for establishment of an exclusive mutualistic relation with their host, as a host gains no advantage by carrying many endosymbionts with identical functions (Kumar et al. 2022). As a result, there seems to be replacements, and even extinction, of several *Coxiella*-like symbionts by *Francisella*-like symbionts in several lineages of ticks (Buysse et al. 2022, Kumar et al 2022). More precisely, in a study by Duron et al. (2017) *Coxiella*-like symbionts were found at a prevalence level of 0.68 in ticks with a single endosymbiont species (ticks can be infected by multiple endosymbionts). However, the prevalence of *Coxiella*-like symbionts was reduced to zero and around by half in ticks that had either *Rickettsiella*-like or *Francisella*-like symbionts as secondary endosymbionts, respectively (Duron et al. 2017). Another interaction has been observed between *Rickettsiella* and *Hamiltonella*, a facultative symbiont, resulting in a negative effect on European populations of the pea aphid *Acyrthosiphon pisum*. Infection with *Rickettsiella* alone had virtually only neutral effects on host fitness, while *Rickettsiella–Hamiltonella* coinfection had negative effects (Tsuchida et al. 2014).

The interactions between other multicellular organisms (other than arthropods) and DIG species are less described in the literature. However, most of these described interactions seem to be pathogenic. For instance, *Legionella* spp., *F. tularensis, C. burnetii*, and *P. salmonis* are all known to cause disease in various mammals and fish species. In these cases, direct and indirect interactions between the various immune systems, tissue cells, and the pathogens occur. For example, *Piscirickettsia* was detected in the digesta and intestinal mucosa of Atlantic salmon. Analysis of the microbiota identified a list of 27 families (e.g. *Lactobacillaceae, Flavobacteriaceae*, and *Tenacibaculum*) with negative associations with *Piscirickettsiaceae* in infected fish, and another set of 10 families with positive associations (Coca et al. 2023). This network analysis suggests that there may be interactions between these bacteria that may produce a metabolic phenotype that favours the virulence of *P. salmonis*, although other factors, such as the age of the host, the impact of other bacteria on the immune system, or certain physico-chemical parameters in the environment of the host might also create those patterns (Coca et al. 2023). In addition, *P. salmonis* and *F. noatunensis* subsp*. noatunensis* were detected at the same time in the gut of *Salmo salar*, suggesting that the interactions between DIGs also occur in other hosts than arthropods (Karlsen et al. 2017). Moreover, DIGs are also being recovered from less well-established and studied animals. For example, *Legionellaceae* and *Coxiellaceae* sequences have been recovered from marine sponges (Yang et al. 2022). Furthermore, nonconventional animal models, such as zebrafish, nematodes, and insect larvae (*Galleria mellonella*), are also being used to study infections of *L. pneumophila* (Leseigneur and Buchrieser 2023). These discoveries point out that DIGs may be associated with a wider range of animals than previously thought, and that certain species may be able to adapt to novel animal species. However, most of these pathogenic interactions have been comprehensively reviewed recently and they will not be discussed in this section (see Liu and Shin 2019, Rozas-Serri 2022).

In summary, DIGs are capable of establishing numerous interactions along the mutualism–parasitism continuum within various environments. However, knowledge of the nature of these interactions is limited, in particular because most research focuses solely on arthropods and/or on DIGs of public health concern (e.g. *L. pneumophila, C. burnetii*, and so on). Furthermore, as the geographical distribution of DIGs is fairly wide (see first section), DIGs associated with unicellular and/or multicellular hosts could differ between certain geographical areas (i.e. tropical rainforests versus more arid regions). Differences in the structure and composition of the bacterial community of certain arthropods and mammals between habitats and geographical sites have already been documented (Namina et al. 2023). These results could therefore indicate the existence of specific interactions between endosymbionts, hosts and the surrounding environment within different ecological niches.

## Concluding remarks

This review highlights links between environmental, evolutionary, and molecular mechanisms of ancient host–endosymbiont interactions along the mutualism–parasitism continuum. The evolution of host–endosymbiont interactions in relation to current environmental trends is important, for example, to predict the potential increase in vector-borne diseases associated with environmental changes. The environmental distribution of DIGs is global, and environmental factors influence their life cycle and virulence. As the global temperature is expected to increase by about 1.5°C by 2050, it is important to take these environmental factors into account. Future work should, therefore, closely examine the influence of environmental factors and the microbiota on the pathogenicity of DIGs. Given the broad global distribution and adaptive capacity of DIG endosymbionts, there is a significant risk of novel pathogens emerging in the coming decades.

Specific molecular mechanisms (T4BSS and biotin biosynthesis) help to facilitate symbiotic interactions across the mutualism–parasitism continuum with specific functions (facilitation of cell adhesion, injection of effectors and virulence factors during host infection, excretion of toxic compounds, and so on). The T4BSS is used by bacteria to inject effector proteins into the host cytoplasm, and effector proteins can be acquired from hosts through HGT. These mechanisms greatly facilitate quick shifts in symbiotic interactions along the parasite–mutualism continuum. In addition, endosymbionts interact not only with their hosts, but also with the microorganisms present in their environment. Indeed, the host microbiota may influence host–bacterial interactions, such as the interactions between *Coxiella* and *Francisella* in ticks. This review highlights the importance of the microbiota in symbiotic interactions across the mutualism–parasitism continuum.

Among the DIG species discussed above, the majority of examples comes from the study of current human (*Legionella, Coxiella*, and *Francisella*) or fish (*Piscirickettsia*) pathogens, for obvious reasons. However, to better understand the emergence of these pathogens, other groups should also be the subject of more research. Among others, the very diverse group related to *Aquicella* (Fig. 1) (Santos et al. 2003), or the unusual intranuclear endosymbiont ‘*Ca*. Berkiella’ (Kidane et al. 2022) are worth studying further.

In conclusion, there is a need for further multidisciplinary research into the evolution of the molecular mechanisms and interactions of endosymbionts with the host microbiota to understand their pathogenic potential. This will allow us to identify the environmental indices and characteristics and community signatures that influence the pathogenicity of these endosymbionts, while developing public health prevention and control strategies.

## Supplementary Material

fuae021_Supplemental_File
