# Supplementary material for: Host–bacteria interactions: ecological and evolutionary insights from ancient, professional endosymbionts
Source: FEMS Microbiol Rev. 2024 Jul 30;48(4):fuae021. doi: 10.1093/femsre/fuae021 (PMC11338181; doi:10.1093/femsre/fuae021)
Supplement: fuae021_Supplemental_File [file fuae021_supplemental_file.docx]

**Supplementary Figure 1.** Global distribution of metagenomic samples from public databases and sample distribution across DIG families with the application of a threshold of 3 DIG-affiliated reads per sample.

**Supplementary Figure 2.** Geographical distribution of DIGs in various environments with the application of a threshold of 3 DIG-affiliated reads per sample. Each panel represents one of the 5 selected DIG families. The sampling location is represented with a dot. Samples are colored according to the biome of origin. (A) Deep-branching Intracellular Gammaproteobacteria, (B) *Coxiellaceae*, (C) *Legionellaceae*, (D) *Piscirickettsiaceae*, (E) *Francisellaceae* and (F) *Fastidiosibacteraceae*.

**Supplementary Figure 3.** Number of DIG-positive samples across various biomes for DIGs with the application of a threshold of 3 DIG-affiliated reads per sample. (A), *Coxiellaceae* (B), *Legionellaceae* (C), *Piscirickettsiaceae* (D), *Francisellaceae* (E) and *Fastidiosibacteraceae* (F).

**Supplementary Figure 4.** Distribution of DIGs across three main environmental factors with the application of a threshold of 3 DIG-affiliated reads per sample. (A) temperature, (B) depth, and (C) salinity, shown for DIGs, *Coxiellaceae*, *Legionellaceae*, *Fastidiosibacteraceae*, *Francisellaceae* and *Piscirickettsiaceae*.
